# Acknowledgment of Reviewers 2025

**DOI:** 10.1177/24731242251411998

**Published:** 2026-04-21

Critical evaluations of articles by expert reviewers make a vital contribution to ensuring the high quality of a journal’s content. The editorial leadership of *Health Equity* is very grateful for the support of the highly qualified peer reviewers who have dedicated their time and effort to reviewing our articles. We would like to show our appreciation by thanking the following individuals for their assistance with the review of articles for the journal in 2025.*

*Data as of November 22, 2025.

Hoda Abdel Magid

Jasmine Abrams

Maria Adams

Brian Adkins

Paris “AJ” Adkins-Jackson

Carlos Aguirre-Nunez

Gloria A. Aidoo-Frimpong

Sirry Alang

Jenifer Allsworth

Alexis Amore

Nafeesa Andrabi

Henry Asante Antwi

Jacob Appel

Mike Armour

Lauren Arrington

Althea Bailey

Ayomide Bankole

Veronica Barcelona

Wendy Barrington

Alex Bauer

Mary Catherine Beach

Ariel Beccia

Gary Beck Dallaghan

Jay Behel

Masoud Behzadifar

April Bell

Meghan Bellerose

Julie Blumenfeld

Emily Boniface

Nicole Boss

Kelly Bower

Heather Bradford

Harry Brown

Danran Bu

Bryan Buckley

Dominique Bulgin

Renee Cadzow

Melanie Canterberry

Lucinda Canty

Liang Chen

MyDzung Chu

Victoria Chu

Kate Clancy

Camille Clare

Emma Clark

Michelle Clausen

Salome Nicole cockern

Akilah Collins-Anderson

Catherine Daily

Elisheva Danan

Sabrina Das

Judith A. Dean

Gina Deck

Jane Desborough

Elizabeth Dickson

LeConte Dill

Brandon Doan

Veronica C. K. Duck

Brenice Duroseau

Hillary Dutton

Judith Eberhardt

Melissa Eggen

Bethany Everett

Nessette Falu

Moshtagh R. Farokhi

Stephanie Fleming

Jill Fleuriet

Konadu Fokuo

Chandra Ford

K. Frazer

Katharine Freire

Liza Fuentes

Lydia Furman

Jose Galvez-Olortegui

Anisha Ganguly

Rocio Garcia-Diaz

Sarah Garrett

Etoi Garrison

Giselle Gerardi

Kimberly Glazer

Keisha Goode

Georgiana Gordon Strachan

Myah Griffin

Lucia Guerra-Reyes

Raul Gutierrez

Amanda Haboush-Deloye

Sarah Haight

Arden Handler

Jing Hao

Idethia S. Harvey

Tyler Harvey

Asha Hassan

Anna Helova

Britany Helton

Anjli Hinman

Xiaolong Hou

Peiyin Hung

Iheoma Iruka

Kortney James

Elizabeth Jarpe-Ratner

Jessica Jensen

Cameron M. Kaplan

J’Mag Karbeah

Jodie Katon

Manorama Khare

Nadeen Khoury

Siribang-on Piboonniyom Khovidhunkit

Heeun Kim

Michelle Ko

Terrence Kominsky

Tom Kottke

Kevin Kovach

Tonette Krousel-Wood

Michael Kuczewski

Elizabeth Lanphier

Kenzie Latham-Mintus

Sam S. S. Lau

Justin A. Lavner

Minh Khoi Le

Yan Li

Jiawei Liu

Jenna LoGiudice

Aurielle Marie Lucier-Julian

Zoe Lucier-Julian

Florence Luhanga

Huabin Luo

Natalie Malone

Luz Adriana Matiz

Morgan Maxwell

Julie McLeod

Anna-Michelle McSorley

Bronwen Merner

Bruce Coffyn Mitchell

Susanna Mitro

Masoud Mohammadnezhad

Diana Montoya-Williams

Isabel Morgan

Christine Morton

Heidi Moseson

Shruti Mutalik

Monika Nayak

Debra Neblett

Peter A. Newman

Ashley Nguyen

Tam Nguyen

Candace Night

Maria Jose Nogueira

Dorian Odems

Rebecca Ofrane

Yasutaka Ojio

Signey Olson

Duniel Ortuño

Amittia Parker

Daniel La Parra-Casado

Susan Kane Patton

Anna Pease

Krystal Pendergraft-Horne

Sayida Peprah-Wilson

Krista Perreira

Liana Petruzzi

Ryan Petteway

Julia Phillippi

Andrew Pickett

Wendy Post

Ana Paula Prata

Maria Pyra

John J. Reynolds-Wright

Korie Rice

David Ring

Candace Robertson-James

Kelley Robinson

Maria Isabel Roldos

Jeff Rose

Micol S. Rothman

Maggie Runyon

Goleen Samari

Bethany Sanders

Gurjit Sandhu

Carolyn Sartor

Golam Sarwar

Rachel Sayler

Davida Schiff

Matthew Schlumbrecht

Jeremy M. Schraw

Shawnda Schroeder

Kathryn Schubert

Aaron T. Seaman

Myesha Senior

Abdolreza Shaghaghi

Rebecca Shasanmi-Ellis

Hafifa Siddiq

Abigail Silva

Rachel Sinkey

Colin M. Smith

Denise C. Smith

Mollee Katherin Steely Smith

Sharla Smith

Stephanie M. Smith

Zoe Smith

Jeremy Snyder

Olga Solomon

Daniel Soto

Maria Soto-Greene

Amanda Spishak-Thomas

Kaitlyn Stanhope

Sarah Stotz

Nancy Sturman

Preeti Sunderaraman

Jessica Tarabay

Tess Thompson

Ariana Thompson-Lastad

Loralei Thornburg

E. Brie Thumm

Ellen L. Tilden

Cecilia Tomori

Carlos Torres

Christine Tucker

Kristin Tully

Korijna Valenti

Clare Viglione

Catherine Vladutiu

Jeannette Marie Wade

Kelly Walker

Jack Wallace

Tess Waxman

Allison Wheeler

Alan Whitaker

John White

Tiffany Williams

Heather Wurtz

Wei Yang

Xin Yin

Lindsay E. Young

Xiao Yu

Lu Zhang

Fei Zhao

Lin Zhu

